# Prevalence of adrenal masses in Japanese patients with type 2 diabetes mellitus

**DOI:** 10.1186/1758-5996-2-71

**Published:** 2010-12-20

**Authors:** Naoki Hiroi, Mariko Sue, Aya Yoshihara, Takamasa Ichijo, Mayumi Yoshida-Hiroi, Mariko Higa, Gen Yoshino

**Affiliations:** 1 Division of Diabetes, Metabolism and Endocrinology, Department of Internal Medicine (Omori), Toho University School of Medicine, Tokyo, Japan; 2 Department of Diabetes and Endocrinology, Saiseikai Yokohamashi Tobu Hospital, Kanagawa, Japan; 3 Department of Internal Medicine, Kawasaki Social Insurance Hospital, Kanagawa, Japan

## Abstract

**Introduction:**

To date, there have been no reports on the prevalence of adrenal masses in type 2 diabetic patients. The present study aimed to evaluate the prevalence of adrenal incidentaloma in type 2 diabetic patients in Japan.

**Subjects:**

We retrospectively evaluated the presence of adrenal masses using abdominal CT scans in 304 type 2 diabetic patients. In those with adrenal masses, we examined the hormone production capacity of the adrenal mass.

**Results:**

Fourteen patients (4.6%) had an adrenal mass. Hormonal analysis identified one case as having subclinical Cushing's syndrome, two with primary aldosteronism. Eleven cases had non-functioning masses.

**Discussion:**

The reported prevalence of adrenal incidentaloma in normal subjects is 0.6-4.0% in abdominal CT scan series. Our results show a relatively high prevalence of adrenal tumors in diabetic patients. On the other hand, the frequency of functional adenoma in diabetic patients is 21.4%, which is similar to that of normal subjects.

**Conclusion:**

Although further studies are needed to evaluate the prevalence of adrenal tumors in diabetic patients, our data suggest that evaluation of the presence of adrenal masses may be needed in patients with type 2 diabetes mellitus.

## Introduction

The detection of clinically silent adrenal masses, known as adrenal incidentalomas, has increased. This has resulted from both technical improvements in abdominal imaging devices and their increased usage. Typically, these techniques include ultrasonography (US), computed tomography (CT scan), and magnetic resonance imaging [1]. As a result, adrenal incidentalomas have become a relatively common finding in clinical practice [2-4]. Many patients with adrenal incidentalomas display altered glucose tolerance, insulin resistance and metabolic syndrome [5,6], though there are no reports on the frequency of adrenal masses in type 2 diabetic patients in Japan. Therefore, we investigated the presence of adrenal tumors in Japanese type 2 diabetic patients.

## Materials and methods

Individuals, presenting to outpatient clinics of the Department of Diabetes, Metabolism and Endocrinology at Toho University Hospital, the Department of Internal Medicine at the Saiseikai Kanagawa-ken Hospital or the Kawasaki Social Insurance Hospital between December 2004 and November 2006, were selected for a retrospective study on the prevalence of adrenal incidentaloma in type 2 diabetic patients. In total, 304 Japanese type 2 diabetic patients (123 women and 181 men; mean age, 61.9 ± 13.2 years [range, 22-89]) were examined in this study. All patients underwent careful clinical examination and none displayed specific symptoms of hypercortisolism. Subjects with a history or clinical evidence of significant secondary hypertension, malignant and/or adrenal tumors, and exogenous glucocorticoid intake were excluded. The main characteristics of the diabetic patients with and without adrenal masses are shown in Table 1.

**Table 1:** General data for the 304 patients with type 2 diabetes mellitus

Diabetic patients (n = 304)	without adrenal mass (n = 290)	with adrenal mass (n = 14)
Age	62.1 ± 13.1	58.7 ± 14.0
Sex (Female/Male)	116/174 (40.0%/60.0%)	7/7 (50.0%/50.0%)
Height (cm)	160.7 ± 9.6	162.3 ± 11.6
Weight (kg)	64.8 ± 16.5	72.7 ± 21.7
Body mass index (kg/m^2^)	24.9 ± 5.6	27.5 ± 7.1
Blood Glucose (mg/dL)	221.1 ± 137.5	168.2 ± 59.8
Glycosylated Hemoglobin (%)	8.5 ± 6.6	7.7 ± 1.4
Urinary C-peptide (μg/day)	46.3 ± 41.2	41.3 ± 15.2
Urinary albumin excretion (mg/g·Cr)	317.7 ± 870.0	437.4 ± 1256.7
Known duration of diabetes (years)	10.3 ± 9.9	12.1 ± 12.1

Data are expressed as means ± SD, number or %.

Abdominal CT scan and US were performed to screen for tumorous lesions including malignant disorders at the time that these diabetic patients presented to our outpatient clinics. Only abdominal CT scan data were selected for this study, because the resolution of CT scans is better than that of US. The imaging characteristics used for the detection of adrenal masses were recorded on the basis of both of a radiologist's and an endocrinologist's description. The diagnosis of cortical adenoma was based on the following CT scan criteria: size >10 mm, smooth margin and homogeneous with a relatively low CT density (≤10 Hounsfield units [HU] on unenhanced CT scan). A non-contrast CT scan attenuation value of 10 HU is a safe cut-off value for differentiating adrenal adenomas from non-adenomas [7,8].

The following hormonal determinations were performed in patients with adrenal masses in order to diagnose the presence of functional adenomas: levels of plasma adrenocorticotropic hormone (ACTH), and serum cortisol, aldosterone, dehydroepiandrosterone-sulfate (DHEA-S) and catecholamines, and plasma renin activity (PRA). Serum cortisol levels were evaluated after an overnight 1 or 8 mg dexamethasone suppression test for the diagnosis of subclinical Cushing's syndrome (SCS). When serum cortisol remained above 3.0 μg/dL using 1 mg dexamethasone or 1 μg/dL using 8 mg dexamethasone, this indicated insufficient suppression. This criterion is recommended by the 'Disorders of Adrenal Hormones' Research Committee of the Japanese Ministry of Health and Welfare [9]. The diagnosis of SCS was made according to the criteria established in the same report [9]. Primary aldosteronism (PA) was diagnosed using the 40 mg furosemide-upright test and the 50 mg captopril-loading test in patients with an aldosterone-renin ratio (ARR) of more than 200. Patients with a PRA level below 1.0 ng/mL/hr 120 minutes after furosemide injection and ARR of more than 300 at 90 minutes after captopril administration were diagnosed as having PA [10-12].

Samples used for measuring hormone levels were all collected at 9 am and centrifuged at 4°C, and plasma and serum were then separated and stored at -20°C until the assays. Plasma ACTH was measured using a commercially available ECLIA kit (Roche Diagnostics, Tokyo, Japan). Serum cortisol and DHEA-S (Diagnostic Products Corp., Tokyo, Japan), and aldosterone (Dinabot Corp., Osaka, Japan) were measured with commercially available RIA kits. Plasma renin activity was determined by RIA kit (TFB, INC., Tokyo, Japan). Catecholamines, adrenaline and noradrenaline were measured by the specific HPLC method [13].

Measurements for diabetic patients with and without adrenal masses were compared using Student's *t *test, and *P *< 0.05 were regarded as significant. Data are expressed as means ± SD.

## Results

An analysis of the age and gender distribution of diabetic patients with adrenal masses showed that they do not differ from diabetic patients without such masses. We determined that there were no significant differences in height, weight, body mass index and known duration of diabetes between these groups. Nor was any significant difference observed in diabetic parameters, such as fasting blood glucose, glycosylated hemoglobin, urinary C-peptide and urinary albumin excretion (Table 1).

Fourteen of the 304 type 2 diabetic patients who were analyzed by CT scan (4.6%; 7 women, 7 men; mean age 58.7 ± 14.0) were found to harbor an adrenal incidentaloma. Adrenal masses were found in the right adrenal gland in seven cases (50.0%), in the left adrenal gland in six (42.9%), and bilaterally in one (7.1%). The mean adrenal mass size was 16.3 ± 5.6 mm (maximum and minimum sizes were 26.0 and 10.0 mm, respectively). In these patients, the mean diameter was 14.3 ± 4.1 mm for non-functioning and 24.3 ± 2.9 mm for functioning masses (*P *< 0.005). Morphologically, all adrenal masses were homogenous with an attenuation value below 10 HU on unenhanced CT scan (mean value, 7.3 ± 2.9; range, 1-10), with neither cysts nor calcification. None of the patients presented with clinical features suggesting any overt adrenal hyper-function (Table 2).

**Table 2:** Characteristics for the 14 patients with adrenal lesions

	ACTH (pg/mL)	COR (mg/dL)	DST		PAC (pg/mL)	PRA (ng/mL/hr)	Confirm exam for PA		Laterality	Size	HU	BP		Diag
			1 mg	8 mg										
	
			COR	COR			Furo-up (PRA)	Capt (ARR)				systolic	diastolic	
Normal range	7.2 ~ 63.3	4.0 ~ 18.3	< 3.0	< 1.0	29.9 ~ 159	0.3 ~ 2.9	< 2.0	< 200						

1	25.4	8.5	0.8		63.5	2.3			Right	10	5	128	76	NF
2	58.3	20.7	2.9		33.3	1.0			Left	10	10	132	80	NF
3	34.8	10.1	< 1.0		88.7	1.4			Left	13	8	130	82	NF
4	25.1	19.3	1.6		67.5	0.9			Right	19	10	134	84	NF
5	80.7	21.7	1.9		104	0.8			Left	10	9	138	88	NF
6	20.6	9.9	2.6		68.7	1.2			Left	16	5	140	80	NF
7	18.8	21.6	2.9		75	1.0			bilateral	21/20	6	130	78	NF
8	77	25.1	< 1.0		69.5	1.1			Right	12	4	134	80	NF
9	29.8	9.2	1.6		83.6	1.7			Right	14	1	148	84	NF
10	28.6	9.5	2.4		60.8	0.9			Left	10	5	140	90	NF
11	31.4	14.2	2.6		100.8	1.0			Right	16	10	126	74	NF

12	7.7	11.7	2.3		227	0.9	0.9	312.0	Right	26	9	140	88	PA
13	16.2	5.5	2.2		128.6	0.2	0.2	416.7	Right	26	10	142	86	PA
14	16.1	21.4	5.3	3.0	88	0.8			Left	21	10	138	84	SCS

Mean ± SD	33.6 ± 22.5	14.9 ± 6.4	2.4 ± 1.1		89.9 ± 45.6	1.1 ± 0.5			Right:7 Left:6 Bilateral:1	16.3 ± 5.6	7.3 ± 2.9	136.6 ± 6.4	82.4 ± 4.7	NF:11 PA:2 SCS:1

Cases 1 to 11 have non-functional adrenal masses, and 12 to 14 have functional adrenal masses including SCS and PA. Data are expressed as means ± SD or number.

ACTH: adrenocorticotropic hormone, COR: corisol, PAC: plasma aldosterone concentration, PRA: plasma rennin activity, DST: dexamethasone suppression test, Furo-upright: frosemide-upright test, Capt: Captopril loading test, HU: Hounsfield unit, NF: non-functional mass, SCS: subclinical Cushing's syndrome, PA: primary aldosteronism

The levels of plasma ACTH (33.6 ± 22.5 pg/mL) and serum cortisol (14.9 ± 6.4 μg/dL) in patients with adrenal masses were within normal ranges. Serum cortisol levels were suppressed after the administration of 1 mg of dexamethasone in cases 1 to 13 but not in case 14 with either 1 mg or 8 mg dexamethasone. The DHEA-S levels were within the age-matched normal range in all but case 14. The aldosterone and PRA levels were 89.9 ± 45.6 pg/mL and 1.1 ± 0.5 ng/mL/hr, respectively. In cases 12 and 13, the ARR was >200, and the furosemide-upright test and captopril loading test were positive but in all other cases the ARR values were <200. We thus diagnosed cases 12 and 13 as having PA, case 14 as having SCS only, and the other 11 cases as having non-functioning adrenal tumors. In both patients with PA, normalization of the ARR and blood pressure decreases were observed after adrenalectomy. However, the SCS case was not treated surgically, because this patient refused adrenalectomy. Since adrenaline and noradrenaline levels were within normal range (the total of adrenaline plus noradrenaline was <500 pg/mL), the possibility of pheochromocytoma was excluded (data not shown).

## Discussion

The mean prevalence of adrenal incidentalomas as determined in autopsy series is 1.9-8.7% [14-16], whereas in CT scan series published in the period from 1982 to 1994, it was 0.6-1.5% [14,15,17-19]. Recently, Bovio et al reported that the prevalence of adrenal masses on abdominal CT scans was 4.4% in Italian patients with high risk of lung cancer, and that of benign adrenal masses was 4.2% [20]. In our study, we observed a relatively high prevalence of adrenal masses (4.6%) in Japanese type 2 diabetic patients. Some reports have shown that patients with adrenal incidentaloma have insulin resistance and metabolic syndrome, although the majority of these masses do not show hormonal hyper-secretion [5,6,21-23]. Terzolo et al demonstrated that non-functioning adenomas have different patterns of steroid secretion [21], and subtle autonomous cortisol secretion might be one of the reasons for the high prevalence of adrenal incidentaloma in type 2 diabetic patients. In addition, recent advances in CT scan technology could be expected to lead to an increased rate of adrenal incidentaloma discovery.

In approximately 70% of cases, adrenal incidentalomas are non-functional adenomas [24]. Investigations of adrenal incidentalomas in Japan have shown the prevalence of functional adrenal adenoma to be approximately 50% [25]. The frequencies of cortisol-producing adenoma and PA in adrenal incidentaloma in Asian populations are 6.0-11.7% and 3.0-4.3%, respectively [25,26]. Herein, a functional adrenal mass in type 2 diabetic patients with adrenal incidentalomas was seen in three out of 14 cases (21.4%), with one case of SCS (7.1%) and two of PA (14.3%). It should be noted that the small number of functional tumors identified in this report may have limited the identification of other types of adrenal functional tumors.

The prevalence of subclinical hyper-cortisolism of adrenal origin in diabetic patients is reported to be 1.1-7.1% [27-29]. In the present study, the prevalence of adrenal SCS was only 0.7% in type 2 diabetic patients. This may be one reason for the low prevalence of adrenal SCS, i.e., that the Japanese diagnostic criteria for SCS were used in this study. The cut-off value of the 1 mg dexamethasone suppression test in the Japanese diagnostic criteria was high, 3.0 μg/dL, in comparison with the diagnostic criteria of the Endocrine Society, for which the cut-off value after 1 mg dexamethasone administration is 1.8 μg/dL [30]. Although levels of cortisol exceeded 1.8 μg/dL after administration of 1 mg dexamethasone in cases 2, 5, 6, 7, 10 and 11, DHEA-S levels were normal. Therefore, the possibility of diabetes seems low in these cases. On the other hand, in case 14 diagnosed with SCS, the DHEA-S level was suppressed with 8.0 μg/dL.

To our knowledge, there are no reports indicating that diabetes induces adrenal incidentaloma, SCS or PA. On the other hand, the pathogenesis of diabetes mellitus is reportedly associated with PA related to impaired insulin secretion due to hypokalemia [31]. Colussi et al demonstrated a direct relationship between aldosterone, insulin resistance and hyperinsulinemia [32,33]. Overproduction of cortisol is reportedly associated with insulin resistance [34], and higher cortisol concentrations are also related to reduced insulin secretion [35]. Therefore, in patients with SCS and PA, diabetes mellitus may develop.

In this study, adrenal masses were found at the same frequency on the right (50.0%) and left sides (42.9%), whereas bilateral masses were detected in one (7.1%) of our cases. A similar lateral distribution of adrenal masses has been reported in CT scan series [25,36,37]. An evaluation of the lateral distribution of adrenal masses using abdominal US showed right predominance [24], whereas Bovio et al reported that masses were detected more frequently in the left adrenal gland [20].

Previously, the sole predictor of the risk of adrenocortical carcinoma was mass size [25,36]. The incidence of malignant adrenal tumors is significantly higher in cases where the size of the masses is greater than 40-50 mm [24,25]. The mean diameter of adrenal masses discovered on CT scans was 16.3 ± 5.6 mm, ranging from 10.0 to 26.0 mm, with no malignancy observed in any of our cases. In addition, these data confirm previous results and show the mean size of functional masses to be significantly higher than that of non-functioning masses [37-39].

## Conclusion

The frequency of functional adrenal masses in Japanese type 2 diabetic patients with such masses was almost the same as that in normal subjects, though a relatively high prevalence of adrenal masses was found in our study. Although further studies are needed to evaluate the frequency of adrenal masses in Japanese type 2 diabetic patients, our data suggest that evaluation for the presence of adrenal masses may be needed in patients with type 2 diabetes mellitus.

## Competing interests

The authors declare that they have no competing interests.

## Authors' contributions

NH participated in the intervention planning and execution of the study, and wrote the manuscript. MS and AY performed the statistical analysis. TI participated in the design of the study. MYH and MH participated in the design of the study and in data collection. GY participated in the design and coordination of the study. All authors have read and approved the final manuscript.
